# LFA-1 Engagement Triggers T Cell Polarization at the HIV-1 Virological Synapse

**DOI:** 10.1128/JVI.01152-16

**Published:** 2016-10-14

**Authors:** Shimona Starling, Clare Jolly

**Affiliations:** Division of Infection and Immunity, University College London, London, United Kingdom; Ulm University Medical Center

## Abstract

HIV-1 efficiently disseminates by cell-cell spread at intercellular contacts called virological synapses (VS), where the virus preferentially assembles and buds. Cell-cell contact triggers active polarization of organelles and viral proteins within infected cells to the contact site to support efficient VS formation and HIV-1 spread; critically, however, which cell surface protein triggers contact-induced polarization at the VS remains unclear. Additionally, the mechanism by which the HIV-1 envelope glycoprotein (Env) is recruited to the VS remains ill defined. Here, we use a reductionist bead-coupled antibody assay as a model of the VS and show that cross-linking the integrin LFA-1 alone is sufficient to induce active T cell polarization and recruitment of the microtubule organizing center (MTOC) in HIV-1-infected cells. Mutant cell lines coupled with inhibitors demonstrated that LFA-1-induced polarization was dependent on the T cell kinase ZAP70. Notably, immunofluorescent staining of viral proteins revealed an accumulation of surface Env at sites of LFA-1 engagement, with intracellular Env localized to a Golgi compartment proximal to the polarized MTOC. Furthermore, blocking LFA-1-induced MTOC polarization through ZAP70 inhibition prevented intracellular Env polarization. Taken together, these data reveal that LFA-1 is a key determinant in inducing dynamic T cell remodeling to the VS and suggest a model in which LFA-1 engagement triggers active polarization of the MTOC and the associated Env-containing secretory apparatus to sites of cell-cell contact to support polarized viral assembly and egress for efficient cell-cell spread.

**IMPORTANCE** HIV-1 causes AIDS by spreading within immune cells and depletion of CD4 T lymphocytes. Rapid spread between these cells occurs by highly efficient cell-cell transmission that takes place at virological synapses (VS). VS are characterized by striking T cell remodeling that is spatially associated with polarized virus assembly and budding at sites of cell contact. Here, we show that the integrin LFA-1 triggers organelle polarization and viral protein recruitment, facilitating formation of the VS, and that this requires the T cell kinase ZAP70. Taken together, these data suggest a mechanism by which HIV-1-infected T cells sense and respond to cell contact to polarize viral egress and promote cell-cell spread. Understanding how cell-cell spread is regulated may help reveal therapeutic targets to specifically block this mode of HIV-1 dissemination.

## INTRODUCTION

Human immunodeficiency virus type 1 (HIV-1) disseminates between T cells either by cell-free infection or by highly efficient cell-cell spread. Cell-cell spread is the predominant mode of HIV-1 dissemination and occurs at virus-induced intercellular contacts known as virological synapses (VS) (1). The HIV-1 VS can be broadly defined as a receptor-containing adhesive junction, characterized by the enrichment of the viral proteins envelope glycoprotein (Env) and Gag in the HIV-infected cell and CD4 and coreceptor (CCR5 or CXCR4) on the target cell, which are collectively polarized at the contact site (1–4). In addition, adhesion molecules, such as lymphocyte function-associated antigen 1 (LFA-1), intercellular adhesion molecule 1 (ICAM-1), and intercellular adhesion molecule 3 (ICAM-3), are also enriched at the VS. Inhibiting either Env-CD4 or LFA-1–ICAM interactions reduces VS formation and cell-cell spread (2, 3, 5), suggesting that both sets of receptor-ligand interactions contribute to driving efficient HIV-1 dissemination by contact-mediated spread. However, an outstanding question remains as to whether integrins, as adhesion molecules, serve simply to stabilize the cell-cell contact, allowing subsequent receptor interactions to drive VS formation, or whether they can induce intracellular signaling that facilitates active VS formation, as is the case for the related human T cell lymphotropic virus type 1 (HTLV-1) VS (6).

Viral budding and assembly occur preferentially at the site of cell contact, resulting in highly efficient and rapid infection of the target T cell (1, 2, 7). Indeed, cell-cell spread of HIV-1 has been shown to be an order of magnitude more efficient than cell-free infection (2, 4, 5, 8–11). Additionally, rapid and focused transfer of virions from one cell to another has been shown to reduce the window of exposure of HIV-1 to neutralizing antibodies and may allow evasion of cellular restriction factors or certain antiretroviral therapies (12–20). Recent intravital microscopy studies have also reported that HIV-1-infected cells show robust migration and form stable cell contacts within a humanized mouse model, providing evidence that cell-cell dissemination could occur *in vivo* (21–23). Thus, cell-cell spread confers many advantages on HIV-1 and potentially plays an important role in viral replication within the host.

Contact of a T cell with an antigen-presenting cell (APC) at the immunological synapse (IS) results in T cell polarization characterized by distinct front and rear morphologies (24–26) and shares some similarities with VS (27). During IS formation, polarization of the microtubule organizing center (MTOC) serves to align the cytoskeleton and to recruit secretory granules and organelles to sites of cell-cell contact (25, 26, 28–31). The VS is associated with striking T cell polarization, with organelles such as mitochondria and the MTOC being found located proximal to viral budding at sites of cell contact (32–34). Furthermore, it has recently been shown that mitochondria are rapidly recruited to VS in response to cell-cell contact and that this supports efficient HIV-1 spread, as well as dynamic calcium flux that is suggestive of activation of T cell signaling at the VS (33). How HIV-1-infected T cells sense and respond to contact and what receptors trigger polarization at the VS remain unclear but have implications for understanding how HIV-1 disseminates between T cells and for the development of novel therapeutics to specifically target this mode of viral spread.

In this study, we developed an antibody-coupled bead assay as a reductionist model of the VS and used the MTOC as a marker to investigate triggers of polarization at VS. Our results identify the integrin LFA-1 as a mediator of T cell remodeling and show that, rather than simply stabilizing cell-cell contacts, LFA-1 signaling via a ZAP70-dependent pathway induces active T cell polarization at the VS. Furthermore, we found that the MTOC is spatially associated with intracellular Env-containing compartments and that LFA-1 engagement induces copolarization of the MTOC and intracellular Env located within the secretory apparatus. Thus, our results address the hitherto outstanding question of how Env is recruited to the VS for viral assembly and provide new insight into cellular processes regulating HIV-1 spread between T cells.

## MATERIALS AND METHODS

### Cell culture and viruses.

The CD4^+^/CXCR4 T cell line Jurkat CE6.1 (from the American Type Culture Collection [ATCC]), LFA-1-negative Jurkat cells (β2.7), LFA-1-negative Jurkat cells expressing wild-type (WT) LFA-1 (β2.7/LFA-1 WT), parental LFA-1-expressing cells (Jn9) (a gift from L. Klickstein, Harvard University) (35, 36), ZAP70-negative Jurkat cells (JP116) (37), ZAP70-reconstituted Jurkat cells (JP116 plus ZAP70 WT) (38), Lck-defective Jurkat cells (JCaM1.6) (39), LAT-negative Jurkat cells (JCaM2) (40), and SLP76-negative Jurkat cells (J14) (41) were cultured in RPMI 1640 suspension cell growth medium (Life Technologies) supplemented with penicillin (100 U/ml), streptomycin (100 μg/ml), and 10% fetal calf serum (FCS) (Labtech). For experiments with primary cells, CD4^+^ target cells were obtained from peripheral blood mononuclear cells (PBMC) isolated from buffy coats from healthy HIV-1-seronegative donors using a Ficoll-Hypaque (Sigma) gradient and activated with 1 μg/ml phytohemagglutinin (PHA) (Sigma) and 10 IU interleukin 2 (IL-2) (Centre for AIDS Reagents, [CFAR], National Institute of Biological Standards and Controls [NIBSC], United Kingdom) in RPMI 1640-20% FCS for 3 days. CD4^+^ T cells were purified by negative selection by magnetic cell sorting (Miltenyi Biotec) according to the manufacturer's instructions and maintained in RPMI 1640-20% FCS, penicillin (100 U/ml), streptomycin (100 μg/ml), and 10 IU IL-2.

The HIV-1 clones pNL4.3 WT (donated by Malcolm Martin and obtained from the AIDS Research and Reference Reagent Program [ARRRP], National Institutes of Health), pNL4.3 ΔCT (donated by Eric Freed), and pNL4.3 ΔEnv and pNL4.3 ΔNef (both donated by Richard Sloan) were used to generate infectious virus by transfecting plasmids into 293T cells using Fugene 6 (Roche). Vesicular stomatitis virus G protein (VSV-G)-pseudotyped virus was generated by transfecting 293T cells using Fugene 6 with pMDG (42) and pNL4.3 WT. The virus was harvested after 48 h and titrated by infectivity assay on HeLa TZM-bl reporter cells (donated by J. Kappes, X. Wu, and Tranzyme Inc. and obtained from the CFAR, NIBSC, United Kingdom), using Bright-Glo (Promega). Additionally, a p24 enzyme-linked immunosorbent assay (ELISA) was performed as previously described (43). Jurkat or primary CD4^+^ cells were spinoculated at 1,200 × *g* for 2 h at room temperature with NL4.3 at a multiplicity of infection (MOI) of 0.1 to 0.2, determined by titration on HeLa TZM-bl cells, and then cultured for 48 to 72 h. For experiments using VSV-G-pseudotyped virus, Jurkat cells were incubated for 4 h with the virus at an MOI of 0.15 before being cultured at 37°C for 48 h. Intracellular Gag staining was performed by fixing cells in 4% formaldehyde and permeabilizing them in BD Perm/Wash buffer (Becton Dickinson), and HIV Gag was detected using the phycoerythrin (PE)-conjugated antibody RC57-RD1 (Coulter). The cells were used for experiments when 80 to 100% were Gag positive.

### Antibody-coupled-bead assays.

The antibodies used for bead labeling were as follows: anti-CD3 clone OKT3 (eBioscience) (44), anti-LFA-1 β_2_ subunit-specific clone L130 (BD Biosciences) (45), anti-LFA-1 α_L_ subunit mouse ascites 25.3.1 (45), and anti-ICAM-1 clone LB-2 (BD Biosciences) (46). Superparamagnetic polystyrene beads (2.8-μm diameter) coated with sheep anti-mouse antibody (DynaBeads M-280 sheep anti-mouse IgG) were coupled with primary mouse antibody (0.1 μg/10^6^ beads; 1/400 for ascites antibodies). The correct coupling of a saturating amount of antibody to the beads was confirmed by flow cytometry. Equal numbers of T cells and beads were incubated in 1% FCS-RPMI on poly-l-lysine-coated coverslips for up to 60 min. For some experiments, T cells were pretreated for 30 min with inhibitors before mixing with the beads (1 μM cytochalasin D, 1 μM nocodazole, or 10 μM piceatannol, all from Sigma-Aldrich). The cell-bead conjugates were fixed using 4% formaldehyde (Sigma-Aldrich) before permeabilization with ice-cold 100% methanol for 5 min or 0.1% Triton X in 5% bovine serum albumin (BSA)-phosphate-buffered saline (PBS) for 20 min at room temperature (only if staining for surface Env). The MTOC was stained with rabbit anti-γ-tubulin (Sigma-Aldrich). For some experiments, intracellular Env was also stained with human anti-gp120 2G12 (Polymun), surface Env was stained with human anti-gp41 (50-69; CFAR), and intracellular Gag was stained with rabbit anti-Gag p24 and p17 (donated by G. Reid and obtained from the CFAR). Primary antibodies were detected with anti-rabbit-conjugated secondary antibodies conjugated to Cy3, Cy5, fluorescein isothiocyanate (FITC), and tetramethyl rhodamine isocyanate (TRITC) (Jackson ImmunoResearch). Coverslips were mounted in Prolong Gold antifade with DAPI (4′,6-diamidino-2-phenylindole) (Life Technologies). To quantify MTOC polarization, single cell-bead conjugates were identified from random fields, and single *xy* slices through the middle of the cell were extracted from *z*-stacks taken through the entire volume of the cell. The cells were manually divided into equal thirds in relation to the position of the bead: proximal, middle, and distal. The MTOC was scored as polarized if it was located in the interface-proximal third of the cell. To quantify viral-protein polarization in response to the antibody-coupled beads, the same process was followed. Cells were scored as polarized if there was a significant accumulation of viral protein in the interface-proximal third of the cell. As an additional alternative measure of polarization, the distance of the MTOC from the site of the cell-bead interface in single cell-bead conjugates was measured manually for each conjugate in micrometers using Huygens Professional version 4.0 software.

### VS preparation and immunostaining.

HIV-1-infected primary CD4 T cells were incubated with an equal number of autologous uninfected target T cells that had been loaded with CellTrace CFSE or calcein violet dye (Life Technologies) according to the manufacturer's instructions. Cell contacts were allowed to form at 37°C in 1% FCS-RPMI on poly-l-lysine-coated coverslips for 60 min. The cells were fixed in 4% formaldehyde for 30 min at 4°C. The cell contacts were permeabilized with ice-cold 100% methanol for 5 min according to published methods (1). The primary antibodies were rabbit anti-γ-tubulin (Sigma-Aldrich), mouse anti-α-tubulin (Sigma-Aldrich), mouse anti-Gag p24 clone 313 (donated by G. Reid and obtained from the CFAR, NIBSC, United Kingdom), human anti-gp120 2G12 (Polymun), mouse anti-giantin 9B6 (Abcam), mouse anti-lamp1 H4A3 (developed by J. T. August and J. E. K. Hildreth and obtained from the Developmental Studies Hybridoma Bank, University of Iowa, Iowa City, IA, USA), mouse anti-EEA-1 clone 14 (BD Biosciences), and rabbit anti-Rab11a (a gift from Scott Lawrence). Primary antibodies were detected with anti-rabbit- or anti-mouse-conjugated secondary antibodies conjugated to Cy3, Cy5, FITC, and TRITC (Jackson ImmunoResearch). Coverslips were mounted in Prolong Gold antifade with DAPI (Life Technologies).

### Microscopy.

Laser scanning confocal microscopy imaging was performed using a Leica SP confocal microscope, and the images were analyzed using the Leica Application Suite Advanced Fluorescence Lite Version 2.2 and Metamorph V7. Alternatively, immunofluorescence (IF) microscopy was performed using a DeltaVision Elite image restoration microscope (Applied Precision) coupled with an inverted Olympus IX71 microscope and a CoolSnap HQ2 camera. Images were acquired and deconvolved with softWoRx 5.0. Processing and analysis were performed using Huygens Professional version 4.0, ImageJ, and Adobe Photoshop 7.

### Colocalization analysis.

Single *xy* slices through the middle of *z*-stacks of HIV-1-infected and uninfected T cell doublets were taken, and the images were deconvolved using softWoRx. To quantify colocalization, the Pearson correlation coefficient values were calculated using the ImageJ coloc 2 plug-in. The plug-in generates a scatter plot showing the pixel-by-pixel intensities of two fluorescent channels, and the Pearson coefficient is generated by dividing the covariances of each channel by their standard deviations. For each staining condition, 20 cell doublets from two independent experiments were analyzed. The Pearson coefficients reported are means and standard errors of the mean (SEM).

### SDS-PAGE and Western blotting.

Uninfected Jurkat cells (3 × 10^6^ to 5 × 10^6^) were lysed in RIPA buffer containing protease inhibitor cocktail (Roche) and PhosStop (Roche). Lysates from equal numbers of cells were loaded on 10% Tris-glycine gels. Proteins were separated by sodium dodecyl sulfate-polyacrylamide gel electrophoresis (SDS-PAGE). Proteins were transferred onto nitrocellulose and blocked in Tris-buffered saline (TBS), 0.1% Tween 20, and 5% skim milk. The blots were probed using rabbit total ZAP70 (Cell Signaling Technology), rabbit total SLP76 (Cell Signaling Technology), rabbit total LAT (Cell Signaling Technology), rabbit total Lck (Cell Signaling Technology), mouse anti-GAPDH (glyceraldehyde-3-phosphate dehydrogenase) (Abcam), or rabbit anti-actin (Sigma). The primary antibody was detected using polyclonal goat anti-rabbit or anti-mouse horseradish peroxidase (HRP) (Dako). Chemiluminescence was detected using the ECL Western blotting system (GE Healthcare). If necessary, blots were stripped and washed before reprobing for additional proteins.

### Statistical analysis.

When comparing normally distributed data under two different conditions, statistical significance was calculated using the Student *t* test. For multiple comparisons, statistical significance was calculated using the parametric analysis of variance (ANOVA) test with Bonferroni correction. Significance was assumed when the *P* value was <0.05. All tests were carried out with GraphPad Prism 6 software.

## RESULTS

### The integrin LFA-1 triggers MTOC polarization in HIV-1-infected T cells.

**FIG 1:**
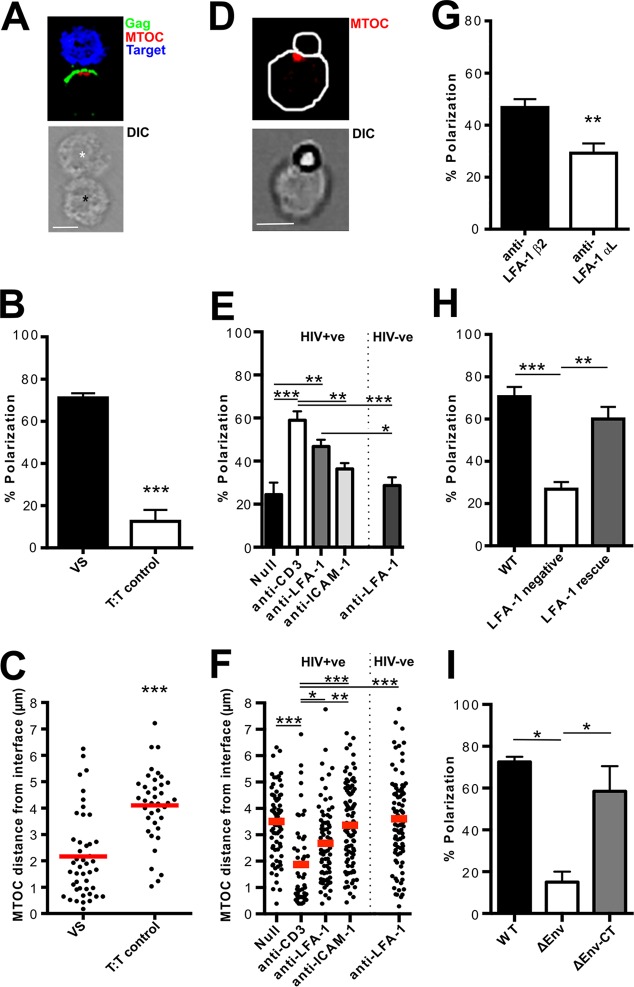
LFA-1 engagement is sufficient to trigger MTOC polarization in HIV-1-infected cells. (A) Using IF analysis, doublets formed from one HIV-1-infected cell (Gag [green] positive; black asterisk) and one uninfected T cell (blue; white asterisk) that showed polarization of Gag to the contact site were identified as VS. The MTOC (γ-tubulin; red) was scored as polarized if it was aligned at the interface-proximal third of the HIV-1-infected cell. DIC, differential interference contrast. (B) Quantification of the percentages of contacts with a polarized MTOC in VS (*n* = 45) and uninfected target-target (T:T) control contacts (*n* = 40). (C) Distance of the MTOC from the cell contact site in VS (*n* = 45) and T-T contacts (*n* = 38). (D) Representative image of an HIV-1-infected Jurkat T cell conjugated with an antibody-coupled bead and stained for the MTOC (γ-tubulin; red). (E) Beads with no antibody (negative control [Null]; *n* = 91) or coupled with anti-CD3 (positive control; *n* = 106), anti-LFA-1 β_2_ subunit (*n* = 193), or anti-ICAM-1 (*n* = 158) were mixed with HIV-1-infected T cells (HIV+ve) or uninfected T cells (HIV-ve), mixed with anti-LFA-1 beads (*n* = 190), and incubated for 60 min. Contacts were scored as polarized if the MTOC fell within the bead-proximal third of the cell. (F) Distance of the MTOC from the cell-bead contact site. (G) As in panel E, but using HIV-1-infected Jurkat cells mixed with beads specific for the LFA-1-β_2_ subunit (*n* = 193) or α_L_ subunit (*n* = 109). (H) Quantification of MTOC polarization in HIV-1-infected mutant or WT Jurkat cells engaged in a VS with an uninfected target T cell. WT Jurkat cells, *n* = 30; LFA-1-negative Jurkat cells, *n* = 30; LFA-1-reconstituted Jurkat cells, *n* = 30. (I) Quantification of MTOC polarization in T cells infected with either WT, ΔEnv, or ΔEnv-CT HIV-1 engaged in contact with an uninfected target T cell. *, *P* < 0.05; **, *P* < 0.01; ***, *P* < 0.001 (three independent experiments). (A and D) Scale bars, 5 μm. The error bars represent SEM. Horizontal bars show the mean.

To determine the plasma membrane triggers of T cell polarization at the VS, HIV-1-infected Jurkat cells were mixed with dye-loaded primary CD4 target T cells at a 1:1 ratio and incubated at 37°C for 60 min before formaldehyde fixation and antibody staining to visualize the MTOC and viral Gag protein; the latter is a well-defined marker of VS formation (1–3, 7, 33). A VS was defined as a doublet composed of an HIV-1-infected Gag-positive cell and an uninfected target T cell, with Gag enriched at the contact site in the infected cell (Fig. 1A). The MTOC is known to be polarized at VS and was used as a marker for organelle polarization within the HIV-1-infected cell (32–34) (Fig. 1A). Contacts were scored as polarized if the MTOC was located in the interface-proximal third in the HIV-1-infected cell. Figure 1A confirms that infected cells forming a VS with an uninfected target cell were significantly more likely to have the MTOC polarized toward the contact site than were two uninfected cells in contact (target-target [T-T] control) (71% polarized VS, 12% polarized T-T control contacts; Student's *t* test; *P* = 0.0001) (Fig. 1B). Additionally, the distance of the MTOC from the site of cell-cell contact was measured in micrometers and was found to be significantly closer in VS than in T-T controls (2.1-μm VS; 4.1-μm T-T control contacts; Student's *t* test; *P* < 0.0001) (Fig. 1C), confirming that the MTOC is localized at the VS.

To identify specific triggers of polarization at VS, antibody-coupled beads were used as a reductionist model of the synapse. Magnetic beads were coupled to antibodies specific for receptors of interest and then incubated with HIV-1-infected Jurkat cells, stained, and analyzed by IF microscopy. Single cell-bead conjugates were scored as polarized if the MTOC was within the interface-proximal third of the cell (Fig. 1D), as described in Materials and Methods. Image analysis software was also used to measure the distance of the MTOC from the cell-bead interface. We tested receptors known to be involved in synapse formation. First, the adhesion molecules LFA-1 and ICAM-1 were examined. Notably, while LFA-1 and ICAM-1 are enriched at the VS (2, 3, 5), whether this interaction simply serves to stabilize cell-cell contacts or whether it plays a more specific role by inducing “outside-in” signaling into cells to facilitate T cell remodeling and VS formation remains unresolved. To investigate the effects of engagement of LFA-1, an antibody specific to the LFA-1 β_2_ subunit (L130) (45) was used. As a positive control for MTOC polarization, we used an antibody against CD3 (OKT3) (44), a component of the TCR complex, since it is well established that the MTOC is recruited to the T cell immunological synapse by engagement of T cell receptor (TCR) (28, 47). Uncoupled “null” beads were used as a negative control. As expected, cross-linking CD3 induced HIV-1-infected T cells to polarize the MTOC to the cell-bead interface (59% of cells polarized with anti-CD3 beads compared to 24% polarized with null beads; one-way ANOVA; *P* < 0.0001). Notably, incubation with anti-LFA-1 β_2_ beads also resulted in significantly more MTOC polarization than incubation with null beads (47% polarized with anti-LFA-1 β_2_ beads and 24% polarized with null beads; one-way ANOVA; *P* < 0.01). Although fewer cells polarized in response to LFA-1 engagement than in response to CD3, the frequencies were not significantly different (*P* > 0.05). In contrast, cross-linking ICAM-1 did not induce significant polarization. Furthermore, uninfected Jurkat cells incubated with anti-LFA-1 β_2_ beads did not show this polarization phenotype, indicating that LFA-1-induced polarization is potentiated by HIV-1 infection (29% of uninfected T cells polarized compared to 47% of HIV-1-infected Jurkat cells; one-way ANOVA; *P* < 0.05) (Fig. 1E).

Measuring the position of the MTOC relative to the cell-bead interface supported these data. Consistent with CD3 engagement providing the strongest stimulus, the MTOC was closest to the interface under these conditions (2 μm from the interface) and furthest away under the null condition (3.5 μm from the interface). In HIV-1-infected Jurkat cells incubated with anti-LFA-1 β_2_ beads, the MTOC was located significantly closer to the cell-bead interface than when incubated with null beads (2.7 μm with anti-LFA-1 compared to 3.5 μm with null beads; one-way ANOVA; *P* < 0.01). Additionally, uninfected Jurkat cells incubated with anti-LFA-1 β_2_ beads had their MTOCs significantly further away from the cell-bead interface, reflecting a nonpolarized phenotype (2.7 μm in HIV-1-infected Jurkat cells compared to 3.6 μm in uninfected Jurkat cells; one-way ANOVA; *P* < 0.01) (Fig. 1F). To provide some context for these distances, the average diameter of a Jurkat cell is approximately 8 μm. We also developed a measurement, termed the “polarization index,” that takes into account the size of a cell and is the ratio between the distance of the MTOC from the interface and the diameter of the cell. When the polarization index was calculated for anti-LFA-1 and anti-ICAM-1 cell-bead conjugates, it mirrored the results seen in Fig. 1F, supporting the observation that LFA-1, but not ICAM-1, was associated with MTOC polarization (data not shown). Together, these data show that LFA-1 engagement alone is sufficient to trigger T cell polarization in HIV-1-infected Jurkat cells.

The integrin LFA-1 is a transmembrane heterodimer composed of two subunits, β_2_ and α_L_ (48). Upon ligand binding, the cytoplasmic tail of β_2_ contributes to outside-in signaling via its interactions with proteins, such as talin and Vav 1 (48). To investigate if LFA-1-associated polarization in HIV-1-infected cells was associated with the active β_2_ component, HIV-1-infected Jurkat cells were incubated with antibody-coupled beads specific to the β_2_ subunit or the α_L_ subunit. Figure 1G shows that cells incubated with the anti-β_2_ beads were significantly more likely to be polarized (47% polarized with anti-β_2_ compared to 29% polarized with anti-α_L_; Student's *t* test; *P* = 0.001), confirming that induction of polarization in HIV-1-infected T cells is mediated by the active β_2_ subunit of LFA-1. As anti-α_L_ beads did not trigger a polarization effect, they were used in further assays as a negative control.

To investigate if LFA-1 was required for T cell polarization in response to cell-cell contact, a cell-based assay using mutant Jurkat derivative cell lines was employed. LFA-1-negative Jurkat cells (Jβ2.7), LFA-1-negative Jurkat cells reconstituted with WT LFA-1 (Jβ2.7/LFA-1 WT), or the parental LFA-1-expressing cells (Jn9) (35, 36) were infected with HIV-1, mixed with dye-loaded primary CD4 target T cells for 60 min at 37°C, and analyzed by IF. VS (as defined above) were scored as polarized if the MTOC was found in the interface-proximal third of the HIV-1-infected T cell. Because LFA-1-negative cells show reduced, but not completely abolished, Gag recruitment and VS formation (5), it was possible to use these cells to score MTOC polarization at sites of cell-cell contact. LFA-1-negative cells showed a significant defect in MTOC polarization at VS (71% of WT compared to 27% of LFA-1 negative cells; one-way ANOVA; *P* < 0.0001) that was rescued by restoring LFA-1 expression (27% in LFA-1-negative compared to 60% in LFA-1-reconstituted cells; *P* < 0.01) (Fig. 1H), confirming the importance of LFA-1 binding for contact-induced polarization in HIV-1-infected T cells at the VS.

In addition to integrins, the HIV-1 envelope glycoprotein (Env) is expressed on the surface of HIV-1-infected T cells, and binding to CD4 on opposing target T cells is a key requirement for VS formation (1–4). To investigate whether Env contributes directly to contact-induced polarization in HIV-1-infected T cells, the cell-based VS assay was again employed. CD4 T cells were infected with WT or VSV-G-pseudotyped ΔEnv HIV-1, mixed with dye-loaded autologous CD4 target T cells for 60 min at 37°C, and analyzed by IF (Fig. 1l). As expected, infected cells not expressing HIV-1 Env (ΔEnv) showed a profound defect in MTOC polarization at infected cell-target cell contacts compared to WT HIV-1 VS, which is consistent with a lack of stable cell-cell contacts and VS formation (1–4). Importantly, T cells infected with a variant of the NL4-3 molecular clone with a 144-amino-acid deletion in the cytoplasmic tail (HIV-1 ΔEnv-CT) (49) showed no defect in MTOC polarization. This deletion leaves only a small portion of Env-CT, anchoring it in the cell membrane and permitting CD4-coreceptor binding via gp120, but removes any potential cytoplasmic signaling domains. Thus, we conclude that direct signaling mediated by the Env-CT is not required to recruit the MTOC, whereas surface Env-gp120 expression is necessary in order to engage CD4^+^ T cells and establish stable contacts for subsequent T cell remodeling mediated by cell-cell receptor interactions.

### LFA-1-induced MTOC polarization is an active process.

**FIG 2:**
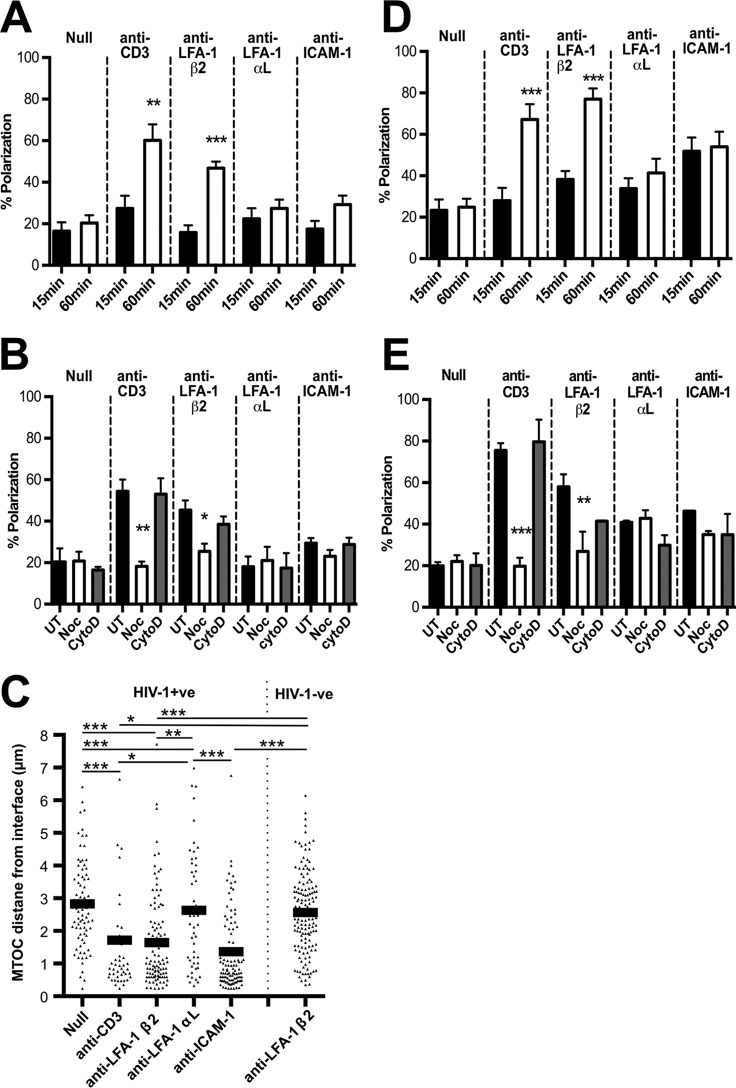
The MTOC is actively and specifically recruited in response to LFA-1 engagement in HIV-1-infected T cells. (A) HIV-1-infected Jurkat cells were incubated with beads with no antibody (Null) (*n* = 58), anti-CD3 (*n* = 51), anti-LFA-1 β_2_ subunit (*n* = 90), anti-LFA-1 α_L_ subunit (*n* = 57), or anti-ICAM-1 (*n* = 112) for 15 or 60 min; fixed; and stained for the MTOC. The MTOC was scored as polarized when it was found in the interface-proximal third of the cell. (B) Quantification of polarization in HIV-1-infected Jurkat T cells pretreated for 30 min with 1 μM nocodazole (Noc) or 1 μM cytochalasin D (CytoD) or left untreated (UT) before being incubated with beads for 60 min and stained as described for panel A. Null, *n* = 90; anti-CD3, *n* = 110; anti-LFA-1 β_2_ subunit, *n* = 120; anti-LFA-1 α_L_ subunit, *n* = 88; and anti-ICAM-1, *n* = 123. (C) HIV-1-infected primary CD4 T cells (HIV+ve) were incubated with beads with no antibody (Null) (*n* = 81) or anti-CD3 (*n* = 45), anti-LFA-1 β_2_ subunit (*n* = 102), anti-LFA-1 α_L_ subunit (*n* = 51), or anti-ICAM-1 (*n* = 107) beads for 60 min. Additionally, anti-LFA-1 β_2_ subunit beads were mixed with uninfected primary CD4 T cells (HIV-ve) (*n* = 142). The distance of the MTOC from the cell-bead interface was measured. (D) HIV-1-infected primary CD4 T cells were incubated with beads with no antibody (Null) (*n* = 111) or anti-CD3 (*n* = 86), anti-LFA-1 β_2_ subunit (*n* = 151), anti-LFA-1 α_L_ subunit (*n* = 134), or anti-ICAM-1 (*n* = 113) beads for 15 or 60 min; fixed; and stained for the MTOC. The MTOC was scored as polarized when it was found in the interface-proximal third of the cell. (E) Quantification of polarization in HIV-1-infected primary CD4 T cells pretreated for 30 min with 1 μM nocodazole (Noc) or 1 μM cytochalasin D (CytoD) or left untreated (UT). The cells were then incubated with beads with no antibody (Null) (*n* = 68) or anti-CD3 (*n* = 44), anti-LFA-1 β_2_ subunit (*n* = 58), anti-LFA-1 α_L_ subunit (*n* = 93), or anti-ICAM-1 (*n* = 102) beads for 60 min and stained as for panel D. *, *P* < 0.05; **, *P* < 0.01; ***, *P* < 0.001 (three separate donors and three independent experiments). The error bars represent SEM.

Next, we sought to confirm that LFA-1-associated polarization in infected cells was an active process indicative of signaling and not simply due to the MTOC already being positioned close to a plasma membrane domain enriched in LFA-1 at which antibody-coupled beads could preferentially bind. To do this, HIV-1-infected Jurkat cells were conjugated with antibody-coupled beads for 15 or 60 min before formaldehyde fixation and staining to visualize the MTOC. Figure 2A shows a time-dependent increase in MTOC polarization in response to LFA-1 β_2_ engagement suggestive of active recruitment, similar to the positive-control CD3. In contrast, HIV-1 Jurkat cells conjugated to anti-LFA-1 α_L_, anti-ICAM-1 beads, or null beads did not show a time-dependent increase in polarization (Fig. 2A). The MTOC is recruited to immune cell contacts in a microtubule-dependent manner (24, 50, 51). Therefore, pharmacological inhibitors were used to disrupt microtubule and actin networks to provide further evidence to support active recruitment. Nocodazole is an inhibitor that depolymerizes microtubules and can prevent MTOC recruitment (52). Cytochalasin D is an inhibitor of actin polymerization and so should not affect MTOC movement (53). HIV-1-infected Jurkat cells were pretreated with either drug for 30 min or left untreated and then incubated with antibody-coupled beads for 60 min before staining to visualize the MTOC. Nocodazole treatment significantly inhibited polarization induced by anti-CD3 and anti-LFA-1 β_2_ beads. In contrast, cytochalasin D had little effect (Fig. 2B). Taken together, these data show that the MTOC is actively trafficked in response to LFA-1 engagement in a manner that requires a functional microtubule network in HIV-1-infected cells.

### LFA-1 induces active MTOC recruitment in HIV-1-infected primary CD4 T cells.

While Jurkat T cells provide a reliable model for HIV-1 infection and VS formation (1–3, 7, 33), we wished to confirm these data using primary T cells. To do this, antibody-coupled bead assays were also performed using HIV-1-infected primary CD4 T cells. Figure 2C shows that, similar to Jurkat cells, anti-LFA-1 β_2_ engagement induced polarization so that the MTOC was located significantly closer to the bead-cell interface than with null beads (1.6 μm with anti-LFA-1 β_2_ beads versus 2.8 μm with null beads; one-way ANOVA; *P* < 0.0001). Additionally, uninfected primary T cells incubated with anti-LFA-1 β_2_ beads were found to have the MTOC significantly further away from the cell-bead interface, which is indicative of HIV-1 infection conditioning cells to polarize, similar to Jurkat T cells (1.6-μm interface distance with anti-LFA-1 β_2_ beads in HIV-1 primary T cells compared to 2.6 μm in uninfected T cells; one-way ANOVA; *P* < 0.0001). Again, anti-LFA-1 α_L_ beads did not induce significant polarization. Interestingly, anti-ICAM-1 beads were associated with a polarized phenotype in primary cells (1.3-μm average distance from the interface) (Fig. 2C). To explore this in more detail, a time course analysis in which cells were conjugated with antibody-coupled beads for 15 or 60 min was performed. As expected, either anti-CD3 or anti-LFA-1 β_2_ induced a significant increase in polarization over time (anti-CD3, 40% increase in polarization over time [Student's *t* test; *P* < 0.0005]; anti-LFA-1 β_2_, 39% increase in polarization over time [Student's *t* test; *P* < 0.0001]) (Fig. 2D). Importantly, engaging ICAM-1 did not increase polarization over time, suggesting that there is no active recruitment of the MTOC. To further analyze MTOC recruitment in HIV-1-infected primary CD4 T cells, the inhibitors nocodazole and cytochalasin D were again used to interfere with microtubule or actin polymerization. As expected, nocodazole abrogated CD3- and LFA-1 β_2_-induced polarization (25% reduction; Student's *t* test; *P* < 0.05) but had no effect on anti-LFA-1 α_L_ or anti-ICAM-1 (Fig. 2E). Together, these data show that engaging LFA-1 actively recruits the MTOC along microtubules to sites of cell contact in HIV-1-infected primary CD4 T cells, in agreement with our observations in Jurkat T cells. Because ICAM-1 engagement is associated with a polarized phenotype but without the requirement for active MTOC trafficking, we conclude that anti-ICAM-1 beads likely bind to a plasma membrane compartment that is enriched in ICAM-1 and already spatially associated with the MTOC; thus, signaling through ICAM-1 does not induce active polarization. This membrane domain may be the uropod that in polarized primary T cells contains the MTOC and is enriched in ICAM-1 and Gag (54).

### LFA-1-induced polarization in HIV-1-infected T cells proceeds through a pathway involving the T cell kinase ZAP70.

**FIG 3:**
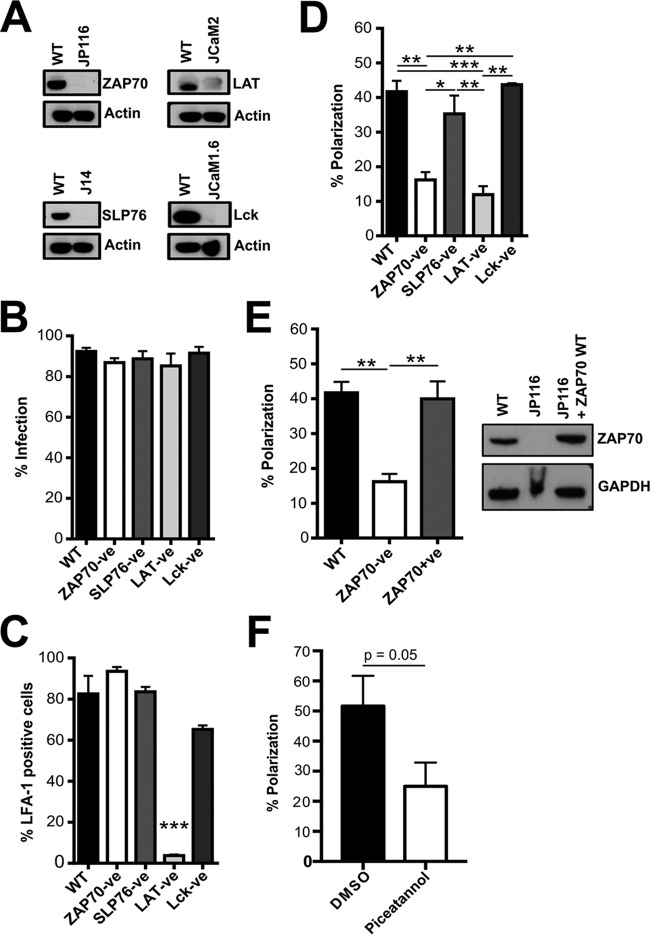
LFA-1-induced polarization proceeds through a pathway involving the T cell kinase ZAP70 in HIV-1-infected T cells. (A) Western blot analysis of uninfected WT Jurkat cells and JP116 (ZAP70-negative), J14 (SLP76-negative), JCaM2 (LAT-negative), and JCam1.6 (Lck-negative) Jurkat cells. (B) WT and mutant Jurkat cells were infected with VSV-G-pseudotyped HIV-1, and the percentage of Gag^+^ HIV-1-infected cells was quantified by flow cytometry. (C) Surface LFA-1 expression on WT, ZAP70-negative (ZAP70-ve), SLP76-negative (SLP76-ve), LAT-negative (LAT-ve), and Lck-negative (Lck-ve) Jurkat cells was quantified by flow cytometry. (D) Quantification of polarized contacts. HIV-1-infected WT (*n* = 55), ZAP70-ve (*n* = 62), SLP76-ve (*n* = 53), LAT-ve (*n* = 53), or Lck-ve (*n* = 64) Jurkat cells were incubated with anti-LFA-1 β_2_ beads. (E) (Left) Quantification of polarized contacts. HIV-1-infected WT (*n* = 47), ZAP70-ve (*n* = 91), or ZAP70+ve (*n* = 72) Jurkat cells were incubated with anti-LFA-1 β_2_ beads. (Right) Western blot analysis of uninfected WT, JP116 (ZAP70-ve), and JP116 ZAP70^+^ (ZAP70+ve) Jurkat cells. (F) HIV-1-infected primary CD4 T cells were pretreated with DMSO (*n* = 29) or piceatannol (*n* = 30) for 30 min before incubation with anti-LFA-1 β_2_ beads and then quantifying polarization by IF analysis as described previously. *, *P* < 0.05; **, *P* < 0.01; ***, *P* < 0.001 (the data are from two independent experiments). The error bars represent SEM.

Active LFA-1-induced T cell polarization is indicative of localized synaptic signaling. To investigate downstream mediators, we took advantage of a panel of Jurkat cell lines defective in key cellular proteins involved in T cell remodeling and organelle polarization at immune cell contacts (55, 56). Specifically, we interrogated ZAP70 (37), SLP76 (41), LAT (40), and Lck (39), which are key regulators of T cell signaling pathways in response to immune cell interactions (57). Western blot analysis confirmed the phenotype of the cells (Fig. 3A), and nearly 100% infection of the cell lines was achieved by using VSV-G-pseudotyped NL4-3 (Fig. 3B). Next, flow cytometry analysis was used to quantify surface LFA-1 expression in the cell lines. With the exception of LAT-negative cells, all the cell lines expressed LFA-1 (Fig. 3C). Next, HIV-1-infected WT or signaling-defective cells were incubated with anti-LFA-1 β_2_ beads for 60 min, and MTOC polarization was scored as previously described. Figure 3D shows that ZAP70-negative cells were significantly impaired in LFA-1 β_2_-induced MTOC polarization to sites of cell-cell contact (one-way ANOVA; *P* = 0.01), but no defect was seen in the absence of SLP76 or Lck. Since LAT-negative cells did not express LFA-1, the failure of these cells to polarize was expected. Restoring expression of ZAP70 in negative Jurkat cells completely rescued LFA-1-induced MTOC polarization in HIV-1-infected cells to WT levels (Fig. 3E). To confirm the importance of ZAP70, HIV-1 primary CD4 T cells were pretreated with piceatannol, a Syk kinase inhibitor that has been shown to specifically inhibit the activation of ZAP70 (58). Treatment with 10 μM piceatannol resulted in reduced polarization of the MTOC at sites of LFA-1 engagement (52% polarized contacts with dimethyl sulfoxide [DMSO] control; 25% polarized contacts with piceatannol treatment; *P* = 0.05) (Fig. 3F). Taken together, these data indicate that LFA-1-induced polarization at the VS proceeds down a pathway requiring ZAP70 in HIV-1-infected T cells.

### LFA-1-induced T cell remodeling is associated with polarization of viral proteins.

**FIG 4:**
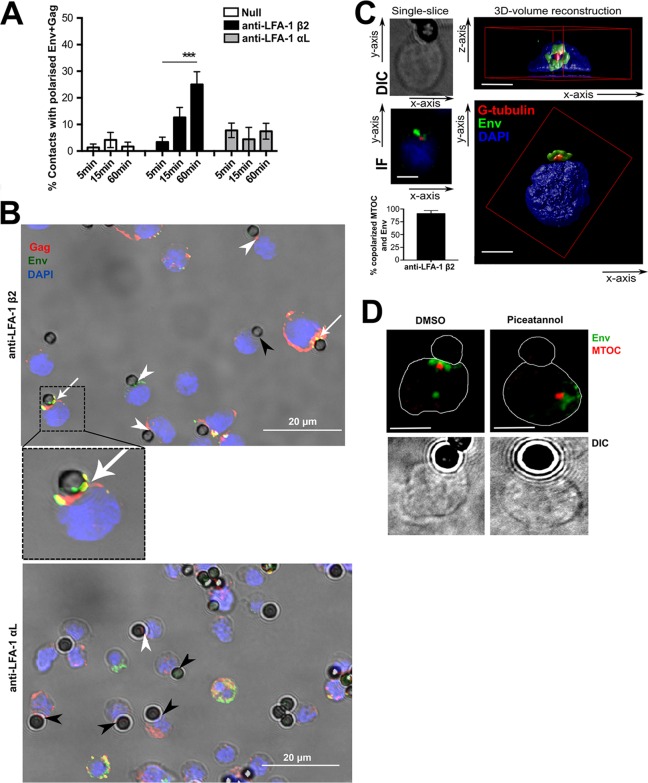
LFA-1-induced T cell remodeling is associated with polarization of viral proteins. (A) Beads with no antibody (Null) (*n* = 225) or coupled with anti-LFA-1 β_2_ (*n* = 215) or anti-LFA-1 α_L_ (*n* = 192) were incubated with HIV-1-infected primary CD4 T cells for 60 min and then stained for intracellular Gag and surface Env. Contacts were scored as polarized if there was a significant accumulation of Env and Gag at the cell-bead interface. (B) (Top) Representative image of HIV-1-infected primary CD4 T cells incubated with anti-LFA-1 β_2_ beads and stained for intracellular Gag and surface Env. (Inset) Example of a cell-bead contact with polarized Env and Gag at the cell-bead interface. (Bottom) As in the top image, but using anti-LFA-1 α_L_ beads. The arrows depict cell-bead contacts with Env and Gag enriched at the interface, the white arrowheads indicate contacts with Env or Gag alone, and the black arrowheads indicate unpolarized contacts. (C) (Left) Representative images of a single section through the middle of an HIV-1-infected T cell–anti-LFA-1 β_2_ bead contact stained for intracellular Env and the MTOC. The MTOC is polarized toward the site of cell contact, and intracellular Env clusters around it. (Right) Two separate images of a 3D reconstruction built from a *z*-stack composed of pictures taken at 0.2-μm intervals through the same cell shown in the IF image on the left. DAPI stain was used to define the top and bottom limits of the cell. The graph on the left shows quantification of contacts with polarized MTOC that also show Env copolarization. (D) HIV-1-infected primary CD4 T cells were pretreated with DMSO or piceatannol for 30 min before incubation with anti-LFA-1 β_2_ beads. Single cell-bead conjugates were identified and scored for MTOC and intracellular Env polarization in relation to the bead interface. The IF images are representative single *xy* slices. Unlabeled scale bars, 5 μm. ***, *P* < 0.001 (three separate donors and three independent experiments). The error bars represent SEM.

To examine the consequences of LFA-1 cross-linking for polarized virus assembly at the VS, antibody-coupled-bead assays were performed using HIV-1-infected primary CD4 T cells coupled with immunofluorescent staining for surface Env (monoclonal antibody [MAb] 50-69) and intracellular Gag. Infected cells were incubated with beads for 5, 15, or 60 min, and contacts were scored as polarized if there was a significant accumulation of viral protein at the cell-bead interface. Unfortunately, the staining conditions required to permeabilize and visualize the MTOC by IF result in poor surface Env staining; therefore, we quantified viral protein polarization at the cell-bead interface in the absence of concomitant MTOC polarization. Despite this, it was striking that anti-LFA-1 β_2_ beads induced a 6-fold increase in the enrichment of surface Env and intracellular Gag to the cell-bead interface over time (4% of contacts at 5 min; 25% of contacts at 60 min; *P* = 0.0003) (Fig. 4A and B). In contrast and as expected, uncoupled null beads induced no recruitment of surface Env or intracellular Gag to the cell-bead interface (Fig. 4A). Likewise, anti-LFA-1 α_L_ beads did not induce viral-protein recruitment (8% of contacts at 5 min; 7% of contacts at 60 min; *P* > 0.05) (Fig. 4A and B).

Little is known about how Env is recruited to the VS. In contrast, Gag has been better studied, and it has been reported that the nucleocapsid domain is required to direct Gag to plasma membrane compartments enriched in uropod markers, termed uropod-directed microdomains (UDMs) (54). As Gag multimerizes, the highly basic region in the matrix domain promotes its targeting to specific UDMs containing PSGL-1, CD43, and CD44 (59). These UDMs have been suggested to carry Gag to the uropods of infected T cells, which forms a prepolarized Gag assembly platform that can participate in VS formation (54). As the intracellular route that Env takes to the VS remains unclear, we decided to further investigate the relationship between Env and T cell polarization induced by LFA-1. Notably, while analyzing intracellular Env staining, we observed that intracellular Env-positive compartments were very frequently clustered around the polarized MTOC. Quantification of this revealed that 91% (±6%) of primary CD4 T cells in contact with anti-LFA-1 β_2_ beads in which the MTOC was polarized to the contact site showed this staining pattern (Fig. 4C). Figure 4C shows *xz* and *xy* images of a three-dimensional (3D)-volume reconstruction of a polarized HIV-1-infected T cell in contact with an anti-LFA-1 β_2_ bead built using *z*-stacks taken 0.2 μm apart in which Env (green) is clearly seen clustered in a compartment around the MTOC (red). We frequently observed this compartment taking a ring-like shape with the MTOC located in the central hole. Importantly, blocking MTOC polarization by inhibiting ZAP70 with piceatannol prevented the recruitment of intracellular Env in response to LFA-1 cross-linking, with Env remaining clustered around the nonpolarized MTOC (Fig. 4D), indicating a functional coupling of MTOC and intracellular Env recruitment to the VS.

### Intracellular Env localizes to a Golgi compartment proximal to the MTOC at the VS.

**FIG 5:**
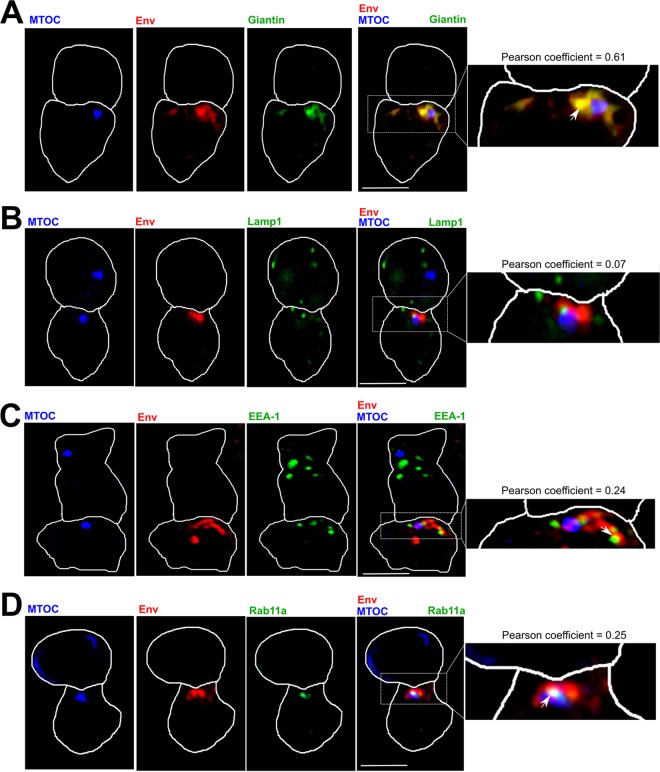
Intracellular Env is localized to a Golgi compartment proximal to the MTOC at the VS. Using IF analysis, doublets formed from one HIV-1-infected primary CD4 T cell (bottom) (intracellular-Env [red] positive) and one uninfected T cell (top) that showed polarization of intracellular Env and MTOC to the contact site were identified as VS. The cells were stained for the Golgi marker giantin (green) (A), the lysosomal marker lamp 1 (green) (B), the early endosome marker EEA-1 (green) (C), or the recycling endosome marker Rab11a (green) (D). The images are single *xy* slices through the middle of the conjugate to locate the MTOC in the infected cell and are representative examples from two independent experiments and two separate donors. The arrows highlight areas of colocalization between Env and the protein of interest. Scale bars, 5 μm. Env colocalization with giantin, lamp 1, EEA-1, or Rab11a was calculated from at least 20 synapses in which the MTOC was localized at the contact site.

To further investigate the MTOC-associated Env-positive compartment, we performed IF microscopy and costained for intracellular organelles. HIV-1-infected primary CD4 T cells were incubated with autologous target cells, fixed, permeabilized, and stained for Env (MAb 2G12); the MTOC (γ- or α-tubulin); and either the Golgi (giantin), lysosome (lamp 1), early endosome (EEA-1), or recycling endosome (Rab11a) compartments, which reflect the intracellular itinerary of newly synthesized and endocytosed Env (60). Analysis of VS with the MTOC polarized to the contact site revealed that Env predominantly colocalized with the Golgi marker giantin in a compartment proximal to the polarized MTOC (Pearson coefficient for Env/giantin = 0.61 ± 0.08) (Fig. 5A). Partial colocalization of Env was also seen with the early endosome marker EEA-1 (Pearson coefficient for Env/EEA-1 = 0.24 ± 0.08) (Fig. 5C) and the recycling endosome marker Rab11a (Pearson coefficient for Env/Rab11a = 0.25 ± 0.05) (Fig. 5D). In contrast, there was little colocalization between Env and the late endosome/lysosomal marker lamp 1 (Pearson coefficient for Env/lamp 1 = 0.07 ± 0.02) (Fig. 5B), consistent with efficient Golgi network retrieval of Env following endocytosis from the plasma membrane (61). Taken together, these data show that intracellular Env is associated close to the MTOC, with most steady-state Env localized to the MTOC-proximal Golgi network that copolarizes to the VS in response to cell-cell contact.

## DISCUSSION

Cell-cell contact at the VS is associated with extensive T cell remodeling, with organelles such as mitochondria and the MTOC reorienting to be proximal to the site of viral egress (32, 33). We have previously shown that calcium-dependent T cell remodeling during cell-cell contact is required for efficient HIV-1 dissemination between T cells. However, the triggers and pathways involved in this process are currently unknown and may have implications for future antiviral strategies to specifically target cell-cell spread. Furthermore, while the requirement for LFA-1/ICAM in VS formation has been demonstrated by many studies (2, 3, 5), the precise role of LFA-1/ICAM at the VS remain unclear. Here, we show that engagement of the T cell integrin LFA-1 is sufficient to actively recruit the MTOC in HIV-1-infected T cells to the VS; that this is mediated by the β_2_ subunit of LFA-1, which is the known signaling component of LFA-1 (48, 62, 63); and that LFA-1-induced T cell polarization requires the kinase ZAP70. In addition to inducing MTOC polarization, LFA-1 cross-linking was also found to recruit viral proteins. Most notably, we found that intracellular Env is localized in a Golgi compartment proximal to the MTOC at the VS and that inhibition of MTOC polarization by pharmacological blockade of ZAP70 signaling also results in loss of intracellular Env clustering at sites of cell contact.

**FIG 6:**
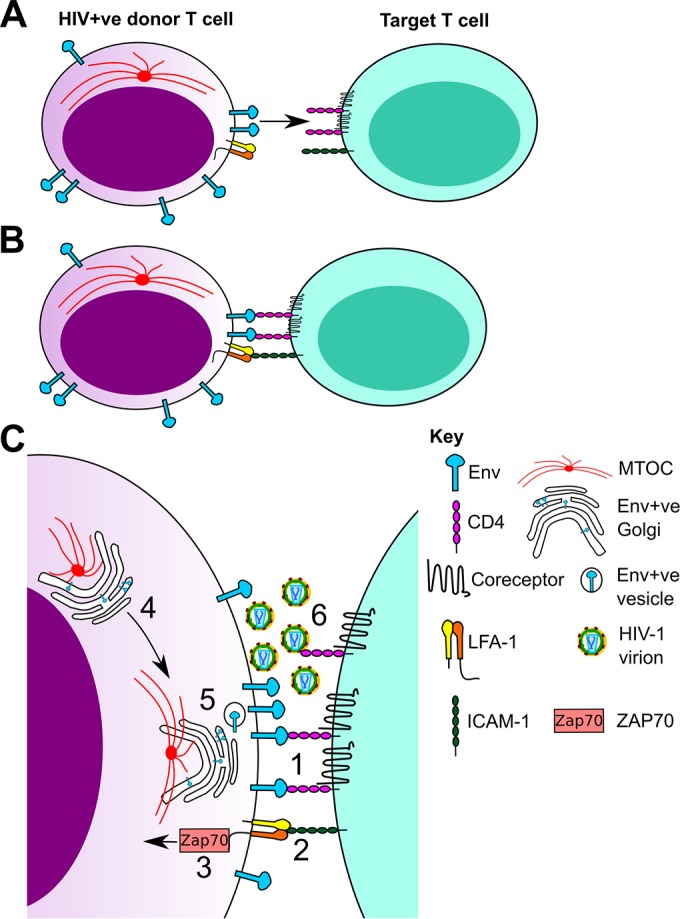
Model of contact-induced polarization that facilitates cell-cell spread at the virological synapse. (A) An HIV-1-infected T cell expressing the envelope glycoprotein (Env) and LFA-1 and an uninfected target cell expressing CD4, coreceptor, and ICAM-1 come into contact. (B) The cell contact drives receptor-ligand interactions between Env-CD4/coreceptor and LFA-1–ICAM-1 that are necessary for cell-cell spread at the VS. (C) Magnified view of events at the contact site. (1) The Env glycoprotein binds to its receptors, CD4 and coreceptor. This allows two CD4 T cells to form a stable contact. (2) On the infected cell, LFA-1 is engaged by its ligand, ICAM-1, which is present on the target cell plasma membrane. This may precede, follow, or be contemporaneous with Env-receptor binding. (3) LFA-1–ICAM binding triggers a signaling event into the infected cell, mediated by the β_2_ subunit of LFA-1 and proceeding through a pathway involving the kinase ZAP70. (4) This signal acts to recruit the MTOC and associated organelles containing intracellular Env. (5) The MTOC moves to be proximal to the cell-cell interface, bringing associated Env-containing components of the secretory apparatus, such as the Golgi complex. This could allow more focused and rapid trafficking of intracellular Env toward a contact zone. (6) The infectious-virus assembly is polarized toward the cell-cell interface, resulting in further elaboration of the VS, directed viral budding, and highly efficient infection of the target cell.

Our data suggest a model (Fig. 6) in which physical contact between an unpolarized HIV-1-infected T cell and a suitable target T cell triggers remodeling that is mediated, at least in part, by LFA-1 signaling into the infected cell through a ZAP70-dependent pathway. This remodeling provides a trigger for T cell polarization and a focal point for the recruitment of MTOC-associated organelles that support cell-cell spread, including the Golgi compartment that resides proximal to the MTOC, thus potentiating delivery of intracellular Env to the plasma membrane (PM) to facilitate full VS formation and polarized virus assembly and budding for rapid infection of target cells by cell-cell spread. While contact-induced T cell remodeling at the VS has been shown to support efficient viral dissemination (1–3, 7, 33), a significant outstanding question remained as to how Env is recruited to sites of cell-cell contact at the VS to support polarized viral assembly and cell-cell spread. During the course of examining MTOC polarization at the VS, we noticed that intracellular Env was frequently found localized around the MTOC in a ring-like structure. Further analysis of intracellular compartments revealed that this was the Golgi compartment and that most intracellular Env clustered around the MTOC was colocalized to the Golgi and to a lesser extent to early and recycling endosomes. Furthermore, LFA-1 binding was able to trigger the polarization of this intracellular Env clustered around the MTOC to the contact zone, while inhibition of MTOC polarization by pharmacological inhibition of ZAP70 blocked this and resulted in Env and the MTOC remaining distal to the VS. In this way, MTOC polarization at the VS may also help facilitate cell-cell spread by recruiting the secretory apparatus and Golgi compartment, which contains the majority of Env (60), allowing specific targeting of Env to the plasma membrane at sites of cell-cell contact. It should be noted that this model of contact-induced T cell polarization is entirely consistent with previous work reporting Gag localization to uropods and uropod-mediated cell-cell contact (54), as it provides an explanation for relocalization of the uropod toward a target cell to form a VS through LFA-1 signaling. Even under conditions where the uropod (which contains the MTOC) had formed a prepolarized Gag-containing platform for cell-cell contact (54), the ability of infected T cells to dynamically remodel, depending on the status of the infected cell and where it forms contact with the target T cell, would afford HIV-1 with the most opportunities to form VS and to spread by highly efficient cell-cell means.

On T cells, the integrin LFA-1 plays an important role in cell arrest during migration and formation of the immunological synapse (64, 65). The β_2_ subunit is involved in outside-in signaling when LFA-1 binds its ligand, ICAM-1 (48). At the immune synapse, it is well known that TCR ligation triggers MTOC recruitment that is required to recruit cellular organelles for polarized secretion (25, 28, 30, 31, 47). However, it has also been demonstrated that under certain chemokine conditions, LFA-1 activation alone can induce fast recruitment of the MTOC and mitochondria to a potential APC (64). We now show that HIV-1 infection of T cells can also serve as a necessary stimulus to prime or condition T cells to undergo contact-induced polarization. The fact that HIV-1-infected T cells are more prone to polarize in response to LFA-1 engagement is consistent with previous reports that describe polarization in HIV-1-infected T cells, but not target T cells, at the VS (32, 33). Here, we have shown that while surface Env expression is necessary for polarization at the VS, consistent with the requirement for Env-CD4 binding in VS formation, the cytoplasmic tail of Env appears to be dispensable, indicating that cytoplasmic signaling directly through Env is not required. Because uninfected CD4 T cells typically form only transient contacts with one another (4), it is likely that Env acts to allow T cells to form sustained homotypic contacts and that this facilitates prolonged engagement of LFA-1 by its ligand, promoting signaling into the cell to induce T cell remodeling. Further work to define the viral protein(s) responsible for conditioning infected cells to polarize would clearly be informative.

LFA-1-induced polarization is indicative of localized signaling, and this study found that the T cell adaptor ZAP70, but not SLP76 or Lck, is required for this process in both HIV-1-infected Jurkat cells and primary CD4 T cells. ZAP70 and Lck have been reported to associate with LFA-1 and are activated by ligand binding (58, 66–68). In support of our results, one other study has also reported that ZAP70-negative cells show a defect in MTOC polarization to the VS (34); however, the molecular mechanisms were less clear, and the triggers for polarization were not examined. Here, we provide some mechanistic insight into the requirement for ZAP70 by linking ZAP70 to a pathway involving cell-cell contact and engagement of LFA-1 that induces T cell remodeling and polarization of organelles and viral proteins, potentially facilitating VS formation and efficient viral spread. In contrast, the role of Lck in T cell polarization is less well defined at both the IS and the VS. At the IS, Lck has been shown by one group (47) to be important for polarization, whereas another group found the related Src family kinase (SFK) Fyn was required for proximal MTOC movement and that Lck was necessary only for centrosome docking at the cell interface (56). Additionally, Fyn has been shown to compensate for an absence of Lck signaling at the IS (69). Further work will be required to determine whether Fyn plays any role in compensating, in part, for the loss of Lck signaling at the VS. Moreover, while we have shown a requirement for LFA-1 in triggering MTOC polarization at the VS, the complexity of multiple receptor interactions that occur during cell-cell contact at the VS means that other cell surface interactions also undoubtedly contribute functionally to VS formation and efficient HIV-1 cell-cell spread through activating synaptic signaling. It would clearly be of interest to further define the molecular details of signaling in recruitment of specific viral and cellular proteins to the VS, as this will provide greater insight into the mechanisms of HIV-1 dissemination and pathogenesis and potentially reveal future strategies to specifically target this mode of HIV-1 dissemination.

## References

[1] Jolly C, Kashefi K, Hollinshead M, Sattentau QJ 2004 HIV-1 cell to cell transfer across an Env-induced, actin-dependent synapse. J Exp Med 199:283–293. doi:10.1084/jem.20030648. 14734528 PMC2211771

[2] Chen P, Hubner W, Spinelli MA, Chen BK 2007 Predominant mode of human immunodeficiency virus transfer between T cells is mediated by sustained Env-dependent neutralization-resistant virological synapses. J Virol 81:12582–12595. doi:10.1128/JVI.00381-07. 17728240 PMC2169007

[3] Rudnicka D, Feldmann J, Porrot F, Wietgrefe S, Guadagnini S, Prevost MC, Estaquier J, Haase AT, Sol-Foulon N, Schwartz O 2009 Simultaneous cell-to-cell transmission of human immunodeficiency virus to multiple targets through polysynapses. J Virol 83:6234–6246. doi:10.1128/JVI.00282-09. 19369333 PMC2687379

[4] Martin N, Welsch S, Jolly C, Briggs JA, Vaux D, Sattentau QJ 2010 Virological synapse-mediated spread of human immunodeficiency virus type 1 between T cells is sensitive to entry inhibition. J Virol 84:3516–3527. doi:10.1128/JVI.02651-09. 20089656 PMC2838118

[5] Jolly C, Mitar I, Sattentau QJ 2007 Adhesion molecule interactions facilitate human immunodeficiency virus type 1-induced virological synapse formation between T cells. J Virol 81:13916–13921. doi:10.1128/JVI.01585-07. 17913807 PMC2168851

[6] Nejmeddine M, Negi VS, Mukherjee S, Tanaka Y, Orth K, Taylor GP, Bangham CR 2009 HTLV-1-Tax and ICAM-1 act on T-cell signal pathways to polarize the microtubule-organizing center at the virological synapse. Blood 114:1016–1025. doi:10.1182/blood-2008-03-136770. 19494354

[7] Hubner W, McNerney GP, Chen P, Dale BM, Gordon RE, Chuang FYS, Li XD, Asmuth DM, Huser T, Chen BK 2009 Quantitative 3D video microscopy of HIV transfer across T cell virological synapses. Science 323:1743–1747. doi:10.1126/science.1167525. 19325119 PMC2756521

[8] Sourisseau M, Sol-Foulon N, Porrot F, Blanchet F, Schwartz O 2007 Inefficient human immunodeficiency virus replication in mobile lymphocytes. J Virol 81:1000–1012. doi:10.1128/JVI.01629-06. 17079292 PMC1797449

[9] Dimitrov DS, Willey RL, Sato H, Chang LJ, Blumenthal R, Martin MA 1993 Quantitation of human immunodeficiency virus type 1 infection kinetics. J Virol 67:2182–2190. 8445728 10.1128/jvi.67.4.2182-2190.1993 PMC240333

[10] Jolly C, Mitar I, Sattentau QJ 2007 Requirement for an intact T-cell actin and tubulin cytoskeleton for efficient assembly and spread of human immunodeficiency virus type 1. J Virol 81:5547–5560. doi:10.1128/JVI.01469-06. 17360745 PMC1900271

[11] Mazurov D, Ilinskaya A, Heidecker G, Lloyd P, Derse D 2010 Quantitative comparison of HTLV-1 and HIV-1 cell-to-cell infection with new replication dependent vectors. PLoS Pathog 6:e1000788. doi:10.1371/journal.ppat.1000788. 20195464 PMC2829072

[12] Titanji BK, Aasa-Chapman M, Pillay D, Jolly C 2013 Protease inhibitors effectively block cell-to-cell spread of HIV-1 between T cells. Retrovirology 10:161. doi:10.1186/1742-4690-10-161. 24364896 PMC3877983

[13] Sigal A, Kim JT, Balazs AB, Dekel E, Mayo A, Milo R, Baltimore D 2011 Cell-to-cell spread of HIV permits ongoing replication despite antiretroviral therapy. Nature 477:95–98. doi:10.1038/nature10347. 21849975

[14] Richardson MW, Carroll RG, Stremlau M, Korokhov N, Humeau LM, Silvestri G, Sodroski J, Riley JL 2008 Mode of transmission affects the sensitivity of human immunodeficiency virus type 1 to restriction by rhesus TRIM5alpha. J Virol 82:11117–11128. doi:10.1128/JVI.01046-08. 18768965 PMC2573261

[15] Jolly C, Booth NJ, Neil SJ 2010 Cell-cell spread of human immunodeficiency virus type 1 overcomes tetherin/BST-2-mediated restriction in T cells. J Virol 84:12185–12199. doi:10.1128/JVI.01447-10. 20861257 PMC2976402

[16] Abela IA, Berlinger L, Schanz M, Reynell L, Gunthard HF, Rusert P, Trkola A 2012 Cell-cell transmission enables HIV-1 to evade inhibition by potent CD4bs directed antibodies. PLoS Pathog 8:e1002634. doi:10.1371/journal.ppat.1002634. 22496655 PMC3320602

[17] Malbec M, Porrot F, Rua R, Horwitz J, Klein F, Halper-Stromberg A, Scheid JF, Eden C, Mouquet H, Nussenzweig MC, Schwartz O 2013 Broadly neutralizing antibodies that inhibit HIV-1 cell to cell transmission. J Exp Med 210:2813–2821. doi:10.1084/jem.20131244. 24277152 PMC3865481

[18] Zhong P, Agosto LM, Ilinskaya A, Dorjbal B, Truong R, Derse D, Uchil PD, Heidecker G, Mothes W 2013 Cell-to-cell transmission can overcome multiple donor and target cell barriers imposed on cell-free HIV. PLoS One 8:e53138. doi:10.1371/journal.pone.0053138. 23308151 PMC3538641

[19] Agosto LM, Zhong P, Munro J, Mothes W 2014 Highly active antiretroviral therapies are effective against HIV-1 cell-to-cell transmission. PLoS Pathog 10:e1003982. doi:10.1371/journal.ppat.1003982. 24586176 PMC3937346

[20] McCoy LE, Groppelli E, Blanchetot C, de Haard H, Verrips T, Rutten L, Weiss RA, Jolly C 2014 Neutralisation of HIV-1 cell-cell spread by human and llama antibodies. Retrovirology 11:83. doi:10.1186/s12977-014-0083-y. 25700025 PMC4189184

[21] Murooka TT, Deruaz M, Marangoni F, Vrbanac VD, Seung E, von Andrian UH, Tager AM, Luster AD, Mempel TR 2012 HIV-infected T cells are migratory vehicles for viral dissemination. Nature 490:283–287. doi:10.1038/nature11398. 22854780 PMC3470742

[22] Sewald X, Gonzalez DG, Haberman AM, Mothes W 2012 In vivo imaging of virological synapses. Nat Commun 3:1320. doi:10.1038/ncomms2338. 23271654 PMC3784984

[23] Sewald X, Ladinsky MS, Uchil PD, Beloor J, Pi R, Herrmann C, Motamedi N, Murooka TT, Brehm MA, Greiner DL 2015 Retroviruses use CD169-mediated trans-infection of permissive lymphocytes to establish infection. Science 350:563–567. doi:10.1126/science.aab2749. 26429886 PMC4651917

[24] Bromley SK, Burack WR, Johnson KG, Somersalo K, Sims TN, Sumen C, Davis MM, Shaw AS, Allen PM, Dustin ML 2001 The immunological synapse. Annu Rev Immunol 19:375–396. doi:10.1146/annurev.immunol.19.1.375. 11244041

[25] Stinchcombe JC, Majorovits E, Bossi G, Fuller S, Griffiths GM 2006 Centrosome polarization delivers secretory granules to the immunological synapse. Nature 443:462–465. doi:10.1038/nature05071. 17006514

[26] Griffiths GM, Tsun A, Stinchcombe JC 2010 The immunological synapse: a focal point for endocytosis and exocytosis. J Cell Biol 189:399–406. doi:10.1083/jcb.201002027. 20439993 PMC2867296

[27] Jolly C, Sattentau QJ 2004 Retroviral spread by induction of virological synapses. Traffic 5:643–650. doi:10.1111/j.1600-0854.2004.00209.x. 15296489

[28] Martin-Cofreces NB, Robles-Valero J, Cabrero JR, Mittelbrunn M, Gordon-Alonso M, Sung CH, Alarcon B, Vazquez J, Sanchez-Madrid F 2008 MTOC translocation modulates IS formation and controls sustained T cell signaling. J Cell Biol 182:951–962. doi:10.1083/jcb.200801014. 18779373 PMC2528574

[29] Stinchcombe JC, Griffiths GM 2007 Secretory mechanisms in cell-mediated cytotoxicity. Annu Rev Cell Dev Biol 23:495–517. doi:10.1146/annurev.cellbio.23.090506.123521. 17506701

[30] Kupfer A, Swain SL, Singer SJ 1987 The specific direct interaction of helper T cells and antigen-presenting B cells. II. Reorientation of the microtubule organizing center and reorganization of the membrane-associated cytoskeleton inside the bound helper T cells. J Exp Med 165:1565–1580. 2953845 10.1084/jem.165.6.1565 PMC2188362

[31] Geiger B, Rosen D, Berke G 1982 Spatial relationships of microtubule-organizing centers and the contact area of cytotoxic T lymphocytes and target cells. J Cell Biol 95:137–143. doi:10.1083/jcb.95.1.137. 6982900 PMC2112358

[32] Jolly C, Welsch S, Michor S, Sattentau QJ 2011 The regulated secretory pathway in CD4 T cells contributes to human immunodeficiency virus type-1 cell-to-cell spread at the virological synapse. PLoS Pathog 7:e1002226. doi:10.1371/journal.ppat.1002226. 21909273 PMC3164651

[33] Groppelli E, Starling S, Jolly C 2015 Contact-induced mitochondrial polarization supports HIV-1 virological synapse formation. J Virol 89:14–24. doi:10.1128/JVI.02425-14. 25320323 PMC4301097

[34] Sol-Foulon N, Sourisseau M, Porrot F, Thoulouze MI, Trouillet C, Nobile C, Blanchet F, di Bartolo V, Noraz N, Taylor N, Alcover A, Hivroz C, Schwartz O 2007 ZAP-70 kinase regulates HIV cell-to-cell spread and virological synapse formation. EMBO J 26:516–526. doi:10.1038/sj.emboj.7601509. 17215865 PMC1783460

[35] Weber KS, York MR, Springer TA, Klickstein LB 1997 Characterization of lymphocyte function-associated antigen 1 (LFA-1)-deficient T cell lines: the alphaL and beta2 subunits are interdependent for cell surface expression. J Immunol 158:273–279. 8977199

[36] Lu CF, Springer TA 1997 The alpha subunit cytoplasmic domain regulates the assembly and adhesiveness of integrin lymphocyte function-associated antigen-1. J Immunol 159:268–278. 9200463

[37] Williams BL, Schreiber KL, Zhang W, Wange RL, Samelson LE, Leibson PJ, Abraham RT 1998 Genetic evidence for differential coupling of Syk family kinases to the T-cell receptor: reconstitution studies in a ZAP-70-deficient Jurkat T-cell line. Mol Cell Biol 18:1388–1399. doi:10.1128/MCB.18.3.1388. 9488454 PMC108852

[38] Blanchard N, Di Bartolo V, Hivroz C 2002 In the immune synapse, ZAP-70 controls T cell polarization and recruitment of signaling proteins but not formation of the synaptic pattern. Immunity 17:389–399. doi:10.1016/S1074-7613(02)00421-1. 12387734

[39] Straus DB, Weiss A 1992 Genetic evidence for the involvement of the lck tyrosine kinase in signal transduction through the T cell antigen receptor. Cell 70:585–593. doi:10.1016/0092-8674(92)90428-F. 1505025

[40] Finco TS, Kadlecek T, Zhang W, Samelson LE, Weiss A 1998 LAT is required for TCR-mediated activation of PLCgamma1 and the Ras pathway. Immunity 9:617–626. doi:10.1016/S1074-7613(00)80659-7. 9846483

[41] Yablonski D, Kuhne MR, Kadlecek T, Weiss A 1998 Uncoupling of nonreceptor tyrosine kinases from PLC-gamma1 in an SLP-76-deficient T cell. Science 281:413–416. doi:10.1126/science.281.5375.413. 9665884

[42] Naldini L, Blomer U, Gallay P, Ory D, Mulligan R, Gage FH, Verma IM, Trono D 1996 In vivo gene delivery and stable transduction of nondividing cells by a lentiviral vector. Science 272:263–267. doi:10.1126/science.272.5259.263. 8602510

[43] Jolly C, Sattentau QJ 2007 Human immunodeficiency virus type 1 assembly, budding, and cell-cell spread in T cells take place in tetraspanin-enriched plasma membrane domains. J Virol 81:7873–7884. doi:10.1128/JVI.01845-06. 17522207 PMC1951303

[44] Adair JR, Athwal DS, Bodmer MW, Bright SM, Collins AM, Pulito VL, Rao PE, Reedman R, Rothermel AL, Xu D 1994 Humanization of the murine anti-human CD3 monoclonal antibody OKT3. Hum Antibodies Hybridomas 5:41–47. 7858182

[45] Springer TA 1994 Traffic signals for lymphocyte recirculation and leukocyte emigration: the multistep paradigm. Cell 76:301–314. doi:10.1016/0092-8674(94)90337-9. 7507411

[46] Bevilacqua MP, Pober JS, Mendrick DL, Cotran RS, Gimbrone MA Jr 1987 Identification of an inducible endothelial-leukocyte adhesion molecule. Proc Natl Acad Sci U S A 84:9238–9242. doi:10.1073/pnas.84.24.9238. 2827173 PMC299728

[47] Lowin-Kropf B, Shapiro VS, Weiss A 1998 Cytoskeletal polarization of T cells is regulated by an immunoreceptor tyrosine-based activation motif-dependent mechanism. J Cell Biol 140:861–871. doi:10.1083/jcb.140.4.861. 9472038 PMC2141749

[48] Hogg N, Patzak I, Willenbrock F 2011 The insider's guide to leukocyte integrin signalling and function. Nat Rev Immunol 11:416–426. doi:10.1038/nri2986. 21597477

[49] Freed EO, Martin MA 1996 Domains of the human immunodeficiency virus type 1 matrix and gp41 cytoplasmic tail required for envelope incorporation into virions. J Virol 70:341–351. 8523546 10.1128/jvi.70.1.341-351.1996 PMC189823

[50] Yi J, Wu X, Chung AH, Chen JK, Kapoor TM, Hammer JA 2013 Centrosome repositioning in T cells is biphasic and driven by microtubule end-on capture-shrinkage. J Cell Biol 202:779–792. doi:10.1083/jcb.201301004. 23979719 PMC3760611

[51] Angus KL, Griffiths GM 2013 Cell polarisation and the immunological synapse. Curr Opin Cell Biol 25:85–91. doi:10.1016/j.ceb.2012.08.013. 22990072 PMC3712171

[52] Burakov A, Nadezhdina E, Slepchenko B, Rodionov V 2003 Centrosome positioning in interphase cells. J Cell Biol 162:963–969. doi:10.1083/jcb.200305082. 12975343 PMC2172857

[53] Eng EW, Bettio A, Ibrahim J, Harrison RE 2007 MTOC reorientation occurs during FcgammaR-mediated phagocytosis in macrophages. Mol Biol Cell 18:2389–2399. doi:10.1091/mbc.E06-12-1128. 17442887 PMC1924806

[54] Llewellyn GN, Hogue IB, Grover JR, Ono A 2010 Nucleocapsid promotes localization of HIV-1 gag to uropods that participate in virological synapses between T cells. PLoS Pathog 6:e1001167. doi:10.1371/journal.ppat.1001167. 21060818 PMC2965768

[55] Kuhne MR, Lin J, Yablonski D, Mollenauer MN, Ehrlich LIR, Huppa J, Davis MM, Weiss A 2003 Linker for activation of T cells, zeta-associated protein-70, and Src homology 2 domain-containing leukocyte protein-76 are required for TCR-induced microtubule-organizing center polarization. J Immunol 171:860–866. doi:10.4049/jimmunol.171.2.860. 12847255

[56] Tsun A, Qureshi I, Stinchcombe JC, Jenkins MR, de la Roche M, Kleczkowska J, Zamoyska R, Griffiths GM 2011 Centrosome docking at the immunological synapse is controlled by Lck signaling. J Cell Biol 192:663–674. doi:10.1083/jcb.201008140. 21339332 PMC3044125

[57] Brownlie RJ, Zamoyska R 2013 T cell receptor signalling networks: branched, diversified and bounded. Nat Rev Immunol 13:257–269. doi:10.1038/nri3403. 23524462

[58] Evans R, Lellouch AC, Svensson L, McDowall A, Hogg N 2011 The integrin LFA-1 signals through ZAP-70 to regulate expression of high-affinity LFA-1 on T lymphocytes. Blood 117:3331–3342. doi:10.1182/blood-2010-06-289140. 21200022

[59] Llewellyn GN, Grover JR, Olety B, Ono A 2013 HIV-1 Gag associates with specific uropod-directed microdomains in a manner dependent on its MA highly basic region. J Virol 87:6441–6454. doi:10.1128/JVI.00040-13. 23536680 PMC3648103

[60] Checkley MA, Luttge BG, Freed EO 2011 HIV-1 envelope glycoprotein biosynthesis, trafficking, and incorporation. J Mol Biol 410:582–608. doi:10.1016/j.jmb.2011.04.042. 21762802 PMC3139147

[61] Groppelli E, Len AC, Granger LA, Jolly C 2014 Retromer regulates HIV-1 envelope glycoprotein trafficking and incorporation into virions. PLoS Pathog 10:e1004518. doi:10.1371/journal.ppat.1004518. 25393110 PMC4231165

[62] Tan SM 2012 The leucocyte beta2 (CD18) integrins: the structure, functional regulation and signalling properties. Biosci Rep 32:241–269. doi:10.1042/BSR20110101. 22458844

[63] Kanner SB, Grosmaire LS, Ledbetter JA, Damle NK 1993 Beta 2-integrin LFA-1 signaling through phospholipase C-gamma 1 activation. Proc Natl Acad Sci U S A 90:7099–7103. doi:10.1073/pnas.90.15.7099. 7688472 PMC47083

[64] Contento RL, Campello S, Trovato AE, Magrini E, Anselmi F, Viola A 2010 Adhesion shapes T cells for prompt and sustained T-cell receptor signalling. EMBO J 29:4035–4047. doi:10.1038/emboj.2010.258. 20953162 PMC3020646

[65] Dustin ML 2007 Cell adhesion molecules and actin cytoskeleton at immune synapses and kinapses. Curr Opin Cell Biol 19:529–533. doi:10.1016/j.ceb.2007.08.003. 17923403 PMC2486492

[66] Morgan MM, Labno CM, Van Seventer GA, Denny MF, Straus DB, Burkhardt JK 2001 Superantigen-induced T cell:B cell conjugation is mediated by LFA-1 and requires signaling through Lck, but not ZAP-70. J Immunol 167:5708–5718. doi:10.4049/jimmunol.167.10.5708. 11698443

[67] Soede RD, Driessens MH, Ruuls-Van Stalle L, Van Hulten PE, Brink A, Roos E 1999 LFA-1 to LFA-1 signals involve zeta-associated protein-70 (ZAP-70) tyrosine kinase: relevance for invasion and migration of a T cell hybridoma. J Immunol 163:4253–4261. 10510363

[68] Soede RD, Wijnands YM, Van Kouteren-Cobzaru I, Roos E 1998 ZAP-70 tyrosine kinase is required for LFA-1-dependent T cell migration. J Cell Biol 142:1371–1379. doi:10.1083/jcb.142.5.1371. 9732296 PMC2149357

[69] Yamasaki S, Tachibana M, Shinohara N, Iwashima M 1997 Lck-independent triggering of T-cell antigen receptor signal transduction by staphylococcal enterotoxins. J Biol Chem 272:14787–14791. doi:10.1074/jbc.272.23.14787. 9169445

